# Polyvalent spherical aptamer engineered macrophages: X-ray-actuated phenotypic transformation for tumor immunotherapy†

**DOI:** 10.1039/d1sc03997k

**Published:** 2021-09-22

**Authors:** Yuanyuan Chen, Peng Gao, Wei Pan, Mingwan Shi, Shujie Liu, Na Li, Bo Tang

**Affiliations:** College of Chemistry, Chemical Engineering and Materials Science, Key Laboratory of Molecular and Nano Probes, Ministry of Education, Collaborative Innovation Center of Functionalized Probes for Chemical Imaging in Universities of Shandong, Institute of Molecular and Nano Science, Shandong Normal University Jinan 250014 P. R. China lina@sdnu.edu.cn tangb@sdnu.edu.cn

## Abstract

Spatiotemporally activatable immune cells are promising for tumor immunotherapy owing to their potential high specificity and low side effects. Herein, we developed an X-ray-induced phenotypic transformation (X-PT) strategy through macrophage engineering for safe and efficient tumor immunotherapy. Without complex genetic engineering, the cell membranes of M0-type macrophages were chemically engineered with AS1411 aptamer-based polyvalent spherical aptamer (PSA) *via* the combination of metabolic glycan labelling and bioorthogonal click reaction. Owing to the superior specificity, affinity and polyvalent binding effects of the high-density AS1411 aptamers, the engineered macrophages could easily recognize and adhere to tumor cells. With further X-ray irradiation, reactive oxygen species (ROS) generated by the Au-based PSA could efficiently transform the accumulated macrophages *in situ* from biocompatible M0 into antitumoral M1 phenotype *via* activating the nuclear factor κB signaling pathway, thereby achieving tumor-specific killing. *In vitro* and *in vivo* experiments confirmed the high tumor recognition and X-ray-induced polarization effect of the engineered macrophages. Compared to natural macrophages, our engineered macrophages significantly inhibited tumor growth in mice even if the radiation dose was reduced by three-fold. We believe this X-PT strategy will open a new avenue for clinical immune cell-based therapy.

## Abstract 2

An X-ray-induced phenotypic transformation strategy (X-PT) through macrophage engineering was developed for safe and effective immunotherapy.

## Graphical abstract

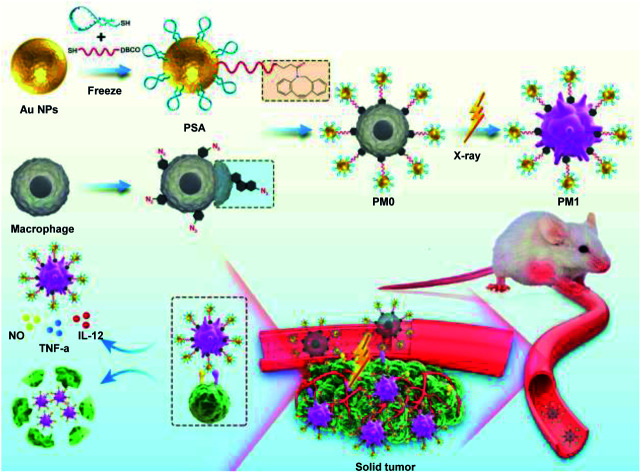

## Introduction

Cell immunotherapy has attracted tremendous attention in recent years and uses reprogrammed immune cells, such as chimeric antigen receptor T (CAR-T) cells and natural killer (NK) cells, as therapeutic agents for cancer treatment.^1–4^ The communication between immune cells and tumor/normal cells decides the therapeutic response and tolerance of the immunotherapy.^5–12^ The high expression of tumor-recognition antibodies on genetically programmed immune cells endow them with better cancer recognition capacity and certain improved therapeutic effects. However, the complicated gene engineering results in a high cost for cell immunotherapy. More importantly, the currently used immune cells are always in a highly active killing state, which could cause severe adverse effects to the patients, due to the continuous secretion of cytokines and inevitable accumulation in healthy tissues.^2^ Hence, methods for enhancing the cell recognition capacity and spatiotemporally controllable activation of the killing effect of immune cells are highly attractive and promising for clinical cancer immunotherapy.

Macrophages, as critical innate immune cells, play important roles in homeostasis and immune responses.^13^ These cells not only phagocytize large particles, such as bacteria, tumor cells, auto-aging and dying cells but also activate the systemic immune response.^14^ Notably, macrophages are highly plastic cells that can transform from one phenotype into others depending on microenvironmental stimuli and signals.^15^ Macrophages can assume opposing phenotypes and functions that can be either tumor-supportive (M2-like cells) or tumoricidal (M1-like cells). Unpolarized M0 and M2 macrophages can be stimulated by reactive oxygen species (ROS) and transform into the M1 phenotype by activating the NF-κB signaling cascade.^16^ M1-type macrophages can kill tumor cells *via* phagocytosis through direct binding or by releasing signaling molecules and cytokines like nitric oxide (NO), ROS, tumor necrosis factor-α (TNF-α) and so on, which could produce sustained cytotoxic effects on tumor cells.^17,18^ Although many efforts have been made to exploit the antitumor activity of macrophages, they are not able to achieve targeted cancer immunotherapy owing to their weak tumor-specific targeting capability.

Aptamers are functional nucleic acids that can mimic antibodies to recognize specific proteins and possess many advantages, including low immunogenicity, good water solubility and high biocompatibility.^19–21^ Engineering micro/nano interfaces with aptamers was indicated to endow these factors with tumor cell recognition/binding abilities.^22–24^ Inspired by the fact that weak interactions can be significantly amplified by polyvalent interactions in nature, we propose polyvalent spherical aptamer (PSA) as promising candidates for cell surface engineering with boosted tumor cell-targeting effects.^25,26^ Owing to the ease of preparation, controllable nucleic acid decoration and excellent radiosensitizing effect of gold nanoparticles (AuNPs) and deep tissue penetration of X-rays,^27–29^ Au-based PSA is suitable for macrophage engineering and phenotype regulation with X-rays.

**Scheme 1:**
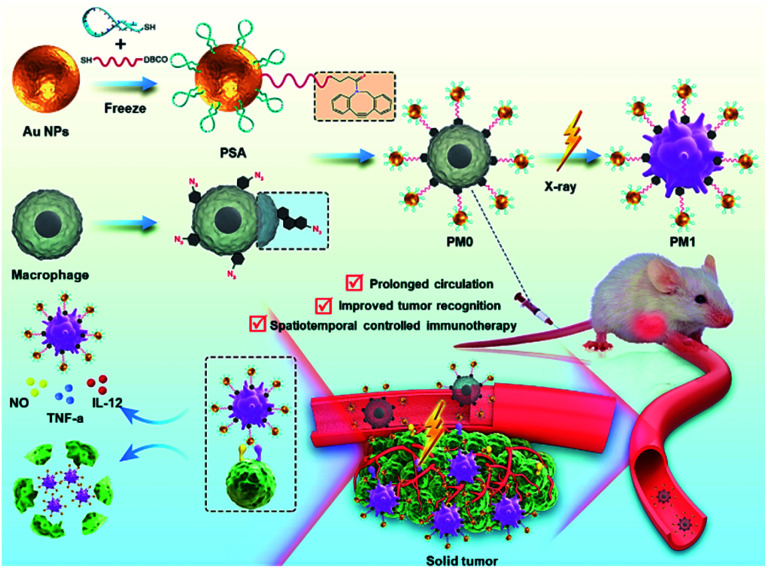
Schematic representation and application of PM0 cells. The synthetic route to PSA-modified M0 macrophage (PM0) and the X-ray-controlled phenotypic transformation of PM0 into PM1 for the on-demand tumor immunotherapy.

Here, we demonstrate an X-ray-triggered phenotypic transformation (X-PT) strategy of engineered macrophages for safe and efficient tumor immunotherapy. Based on metabolic glycan biosynthesis and click reaction, biocompatible M0 macrophages were chemically functionalized with Au-based PSA to obtain engineered macrophages (PM0) without genetic alteration. The low immunogenicity and physiological stability of PSA endowed PM0 cells with improved circulatory performance *in vivo*. Benefiting from the specific recognition and polyvalent binding effects of PSA with tumor cell receptors, PM0 cells exhibit a significantly better recognition effect towards tumor cells. When injected back into tumor-bearing mice, PM0 cells could efficiently target and accumulate in tumor tissues with different tumor volumes. With further ultralow-dose X-ray irradiation, ROS produced by PSA could further activate the NF-κB pathway to induce the transformation of the engineered macrophages into antitumor M1 phenotype, which exhibited strong phagocytic and antitumor cytokine secretion effects, thereby mediating specific and efficient tumor treatment. This developed X-PT is expected to be a versatile strategy for clinical cancer immunotherapy (Scheme 1).

## Results and discussion

### Preparation and characterization of polyvalent spherical aptamer (PSA)

**Fig. 1:**
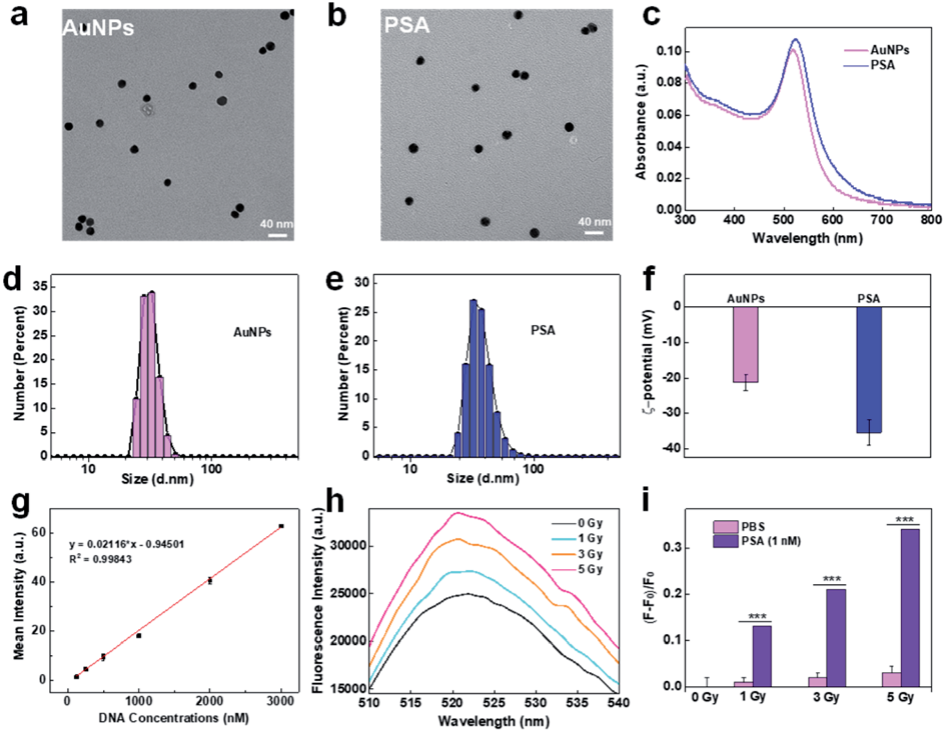
Synthesis and characterization of PSA. TEM images of AuNPs (a) and PSA (b); the UV-vis absorption spectra of AuNPs and PSA (c); particle size of AuNPs (d) and PSA (e) by DLS analysis; zeta potential analysis (f); standard linear calibration curve of DNA by NanoDrop analysis (g); the fluorescence intensity of DCFH in PSA solution with different treatments (h); the relative enhancement of DCFH fluorescence in PSA or PBS solution with different doses of X-ray irradiation, where *F*_0_ is the intrinsic fluorescence without X-ray irradiation, respectively (i).

PSA was synthesized by immobilizing two types of thiol-modified DNA chains (DNA1 and DNA2) onto gold nanoparticles (AuNPs) *via* the freezing method.^24^ Due to their ease of modification, good biocompatibility and intrinsic radiosensitive activities, AuNPs are suitable as radiosensitive therapeutic agents.^30,31^ The 13 nm citrate-capped AuNPs were prepared according to a previously reported method.^32,33^ DNA1 is a thiol-functionalized AS1411 aptamer that can enhance the recognition and killing efficiency of immune cells through its nucleolin-targeting effect. DNA2, labeled with thiol at 5′-end and dibenzocyclooctyne (DBCO) at 3′-end, was introduced as a linker between the PSA and macrophages because DBCO can specifically and spontaneously react with azide. The corresponding DNA sequences are listed in Table S1.† Transmission electron microscopy (TEM, Fig. 1a and b) images and dynamic light scattering (DLS) analysis (Fig. 1d and e) showed that AuNPs exhibited uniform spherical morphology with an average diameter of ∼13 nm, and PSA showed slight increases in particle size. The UV-vis spectrum showed that the maximum absorption peaks of AuNPs and PSA shifted from 519 nm to 524 nm, indicating successful functionalization with DNA1 and DNA2 (Fig. 1c). The changes in zeta potential (Fig. 1f) further confirmed this result. Additionally, the DNA density of the PSA was calculated to be 90 ± 9 per AuNPs by NanoDrop analysis (Fig. 1g). Among them, 85 ± 5 DNA1 and 10 ± 3 DNA2 were labeled on each AuNPs according to the fluorescence standard curves (Fig. S1†). Finally, the radiosensitive properties of the PSA was evaluated. AuNPs are high Z nanomaterials that can not only increase radiation energy deposition but also promote ROS generation through photocatalysis. We chose 2′,7′-dichlorodihydrofluorescein (DCFH) as a fluorescent indicator to determine the ability of PSA to generate ROS. As shown in Fig. 1h and i, the green fluorescence signal grew brighter in a dose-dependent manner at a PSA concentration of 1 nM. Under the same dose of X-ray irradiation, the relative fluorescence enhancement in PSA solution was much higher than that in the PBS group, which indicated a good radiosensitization effect.

### Engineering macrophages with PSA

PSA was subsequently used for macrophage engineering based on metabolic labeling and bioorthogonal click reactions.^34,35^ Specifically, macrophages were first incubated with *N*-azidoacetylmannosamine (Ac_4_ManNAz) for 24 h to decorate the glycoproteins on the cell surface with azide tags. Then, DBCO-labeled PSA was covalently conjugated with these azido residues to obtain the final engineered macrophages, which were named PM0. The methyl thiazolyl tetrazolium (MTT) assay revealed that neither Ac_4_ManNAz nor PSA exhibited obvious toxicity to macrophages within 24 h, even at concentrations of Ac_4_ManNAz up to 100 μM or concentrations of PSA up to 1.5 nM, suggesting the excellent biocompatibility of the engineering strategy (Fig. S2†). Accordingly, 50 μM Ac_4_ManNAz and 1.0 nM PSA were chosen for the following study. Furthermore, the effects of macrophage engineering on cell phenotype were also investigated *via* flow cytometry. As shown in Fig. S3,† similar to that of unpolarized cells, no significant expression of CD80 (M1 marker) was observed after Ac_4_ManNAz or PSA treatment, indicating little effect of engineering on macrophage phenotype. When Ac_4_ManNAz-treated or PSA-treated cells were further incubated with 0.2 mM Mn^2+^ for 24 h, the population of CD80+ M1 cells was markedly increased, further illustrating that the modification strategy had little effect on the cell phenotype and that PSA-engineered macrophages could efficiently polarize from the M0 to the M1 phenotype in response to Mn^2+^, similar to normal macrophages.

### Characteristics of PM0 cells

**Fig. 2:**
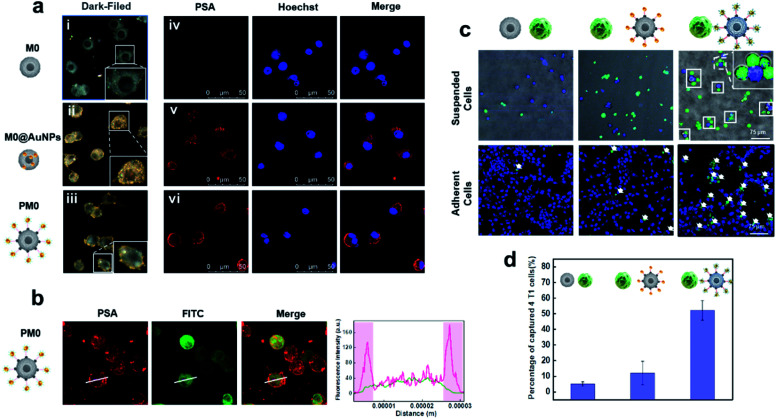
Characteristics of PM0 (a). Dark field images (i–iii) and CLSM images (iv–vi) of macrophages after different modifications; CLSM images of PM0 (b) and the quantification of fluorescence intensity of the line scanning profiles in the corresponding confocal images in b (inset); CLSM images of the interaction between macrophages and 4T1 cells (c). 4T1 cells were prelabeled with Hoechst and macrophages were prelabeled with DiO; quantitative analysis of captured 4T1 cells in suspended cells (d).

To evaluate the cell membrane anchoring performance of PSA, dark-field microscopy (DFM) and confocal laser scanning microscopy (CLSM) were utilized to observe the distribution of PSA on cells. As shown in Fig. 2a and b, DFM imaging of cells treated with PSA showed strong membrane localization of AuNPs (iii), while cells treated with the inactive probe (lacking DNA2 modification) had an average distribution of AuNPs on the cells (ii). CLSM imaging further confirmed that distinct fluorescence was observed on the surface of cells only in the PSA-treated group (vi), and the control group of M0@AuNPs without membrane anchoring properties could be easily taken up by cells (v). These results suggested that macrophages incubated with Ac_4_GalNAz could successfully express azide groups on the cell surface and be chemoselectively decorated with DBCO-modified PSA.

### Tumor cell selectivity of PM0 cells

To evaluate whether the PSA engineering strategy could enhance the communication between macrophages and cancer cells, the selective targeting capability of the AS1411 aptamer was first investigated in tumor cells and normal cells. Cy5-labeled PSA was incubated with 4T1 cells (murine breast carcinoma cells) or TC-1 cells (mouse lung epithelial cells) for 1 h and then visualized by CLSM. Obvious red fluorescence signals were localized on the membrane of 4T1 cells, while negligible fluorescence intensity was observed in TC-1 cells, indicating that PSA conjugated with the AS1411 aptamer could specifically target tumor cells (Fig. S4†). Next, we used CLSM to visually observe the interaction between PM0 cells and cancer cells using suspension cells, in which M0 cells were prelabeled with DiO dye and 4T1 cells were prelabeled with Hoechst. As shown in Fig. 2c and d, compared to that of unmodified macrophages or AuNPs-incubated macrophages, a large number of PM0 clusters appeared to be attached to 4T1 cells, and the percentages of captured 4T1 cells were approximately 5%, 12% and 52% for macrophages, AuNPs-incubated macrophages and PM0 cells, respectively. Additionally, the interactions between PM0 cells and cancer cells were also studied using adherent cells (Fig. 2c). 4T1 cells were preincubated for 24 h in confocal dishes, and then differentially treated macrophages were added. After coincubation for an additional 1 h, the 4T1 cells were washed with PBS to remove the unattached macrophages. CLSM images showed that more PM0 cells adhered to the 4T1 cells than those in the other two groups, further suggesting that PSA-engineered macrophages possessed significant affinities for tumor cells. Therefore, the PSA-engineering strategy could effectively improve the communication between macrophage cells and cancer cells through polyvalent interactions.

### Stimulated differentiation of PM0 cells from the M0 to M1 phenotype

**Fig. 3:**
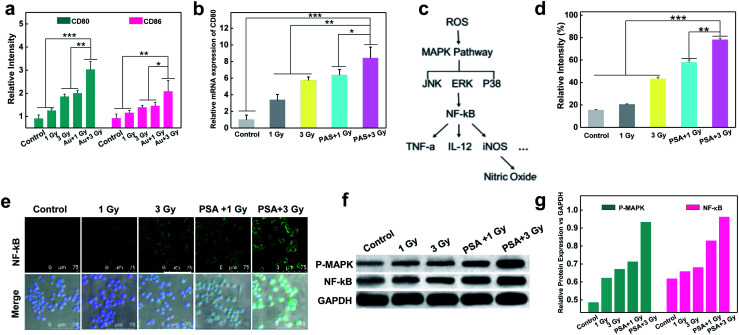
Stimulated differentiation of PM0 cells from the M0 to M1 phenotype and the mechanism. Corresponding relative expressions of the CD80 and CD86 in macrophages with different treatments by immunofluorescence staining (a); relative mRNA expressions of CD80 in different treated-macrophages by q-PCR analysis (b); schematic illustrating the mechanism of ROS-activated M1 signal transduction (c); intracellular ROS generation in different treated macrophages by flow cytometry analysis (d); immunofluorescent staining showing NF-κB expression in macrophages that received various treatments (e); protein levels of NF-κB and P-MAPK in different treated macrophages determined by western blotting (f). Corresponding relative expressions in f (g); statistical analysis was performed by two-tailed paired sample Student's *t*-tests (*p < 0.05, **p < 0.01, ***p < 0.001).

To test whether X-rays could polarize M0 macrophages to the antitumoral M1 phenotype, the expression of M1 markers (CD80 and CD86) on macrophages with different treatments was measured. As shown in Fig S5† and 3a, a dose-dependent increase in green fluorescence signals suggested that X-ray irradiation could promote macrophage transition from the M0 to the M1 phenotype. Notably, the expression of CD80 on PM0 cells after 1 Gy X-ray irradiation was similar to that in natural macrophages with 3 Gy X-ray irradiation, and the highest expression of CD80 was observed on PM0 cells with 3 Gy X-ray irradiation, illustrating the efficient M1 polarization induced by the combination of PSA modification and X-ray irradiation. These results were consistent with those of CD80 mRNA levels, as determined by q-PCR analysis (Fig. 3b). Moreover, changes in the expression levels of CD86 on macrophages after different treatments showed the same trend as that of CD80 by CLSM analysis. Flow cytometry further showed that the percentages of CD80- and CD86-positive cells were significantly increased in the PSA+3 Gy-treated group (Fig. S6†). These results proved that PSA-engineered macrophages could be effectively activated into the M1 antitumoral phenotype by mild X-ray irradiation.

### The mechanism of macrophage polarization

The molecular mechanism by which unpolarized M0 macrophages were stimulated to form the M1 phenotype was subsequently investigated. It is known that ROS play an important role in macrophage polarization *via* the activation of mitogen-activated protein kinase (MAPK) and nuclear factor κB (NF-κB) signaling cascades^36,37^ (Fig. 3c). Thus, the total ROS generation in macrophages after different treatments was examined using 2,7-dichlorofluorescein diacetate (DCFH-DA) as an indicator.^38–41^ As shown in Fig. S7† and 3d, under the same dose of X-ray irradiation, the fluorescence intensity in PM0 cells was higher than that in untreated cells, which may be attributed to the fact that PSA engineering facilitated ROS generation in macrophages. Next, the activation of NF-κB was studied *via* CLSM (Fig. 3e). The images suggested that the expression of NF-κB was evidently upregulated in the PM0+3 Gy group compared with the other control groups, demonstrating that NF-κB could be effectively activated and successfully translocated from the cytoplasm to the cell nuclei. Additionally, the western blot results further confirmed that the protein levels of MAPK and NF-κB were significantly elevated in PM0 cells after X-ray irradiation (Fig. 3f and g). These data proved that Au-based PSA acts as radiosensitizers to enhance intracellular ROS generation under mild X-ray irradiation, which could further transform M0 macrophages into the M1 phenotype by activating the M1 signaling pathway.

### The enhanced cytotoxicity of PM0 cells

**Fig. 4:**
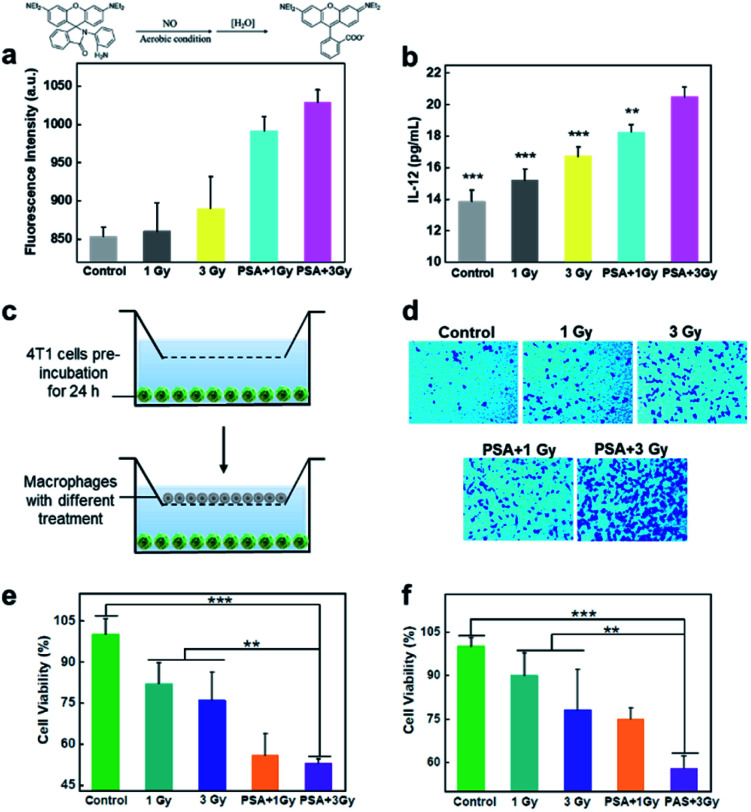
Quantification of cytokine secretion and the enhanced cytotoxicity of PM0. Detection of NO content in the upper culture medium of macrophages treated with different conditions (a); IL-12 secretion from different treated-macrophages as determined by ELISA analysis (b); sketch depicting the chemotaxis assay of macrophages (c); images of macrophages transported in the lower chamber of the transwell system (d); cell viabilities of 4T1 tumor cells incubated with different treated macrophages (e) or incubated with upper culture medium of macrophages treated with different conditions (f).

Compared with unpolarized macrophages, M1 cells can produce nitric oxide (NO), interleukin-12 (IL-12) and other cytokines to exert effective anticancer effects.^42,43^ Thus, we first measured NO generation using a luminescent probe.^44^ After macrophages were subjected to different treatments for 12 h, the culture medium in the different groups was collected and centrifuged. Then, the fluorogenic NO probe was incubated with the culture medium, and the fluorescence intensity was recorded. As shown in Fig. 4a, no obvious fluorescence signal was observed in the control groups, while enhanced fluorescence intensity was noted in the X-ray-irradiated group, demonstrating NO generation. Notably, in response to 3 Gy irradiation, the amount of NO generated by PM0 cells showed a 1.4-fold increase compared with that in untreated cells. In addition, the enzyme-linked immunosorbent assay (ELISA) results showed that the expression of IL-12 was significantly increased after the integration of PSA and X-ray treatment of macrophages (Fig. 4b). These results indicated that activated PM1 cells had great potential to kill tumor cells and induce the immune response. Next, macrophage chemotaxis toward cancer cells was investigated *via* a Transwell migration assay.^45^ The lower compartment was preincubated with 4T1 cells for 24 h, while differentially treated macrophages were seeded in the upper chamber. Following coincubation for 6 h at 37 °C, macrophages that crossed the Transwell into the lower chamber were stained with crystal violet and analyzed under a fluorescence microscope. The resultant data showed that few macrophages passed through the chamber in the control group, indicating their weak migration toward 4T1 cells (Fig. 4d). When macrophages were modified with PSA and irradiated with 3 Gy X-ray, a large number of macrophages were observed in the lower chamber, illustrating the improved chemotaxis of PM1 cells toward tumor cells. Finally, to determine whether activated PM1 cells exhibited increased tumoricidal activity *in vitro*, 4T1 cells were cocultured with differently treated macrophages (Fig. 4e) or the corresponding culture medium (Fig. 4f). The results of the MTT assay showed that when the macrophages underwent both PSA modification and X-ray irradiation, the cells and culture medium could significantly reduce the viability of 4T1 cells compared with that in any of the other control groups, which demonstrated that PSA-engineered macrophages possess strong phagocytic abilities and that their secretions also showed robust anticancer effects.

### *In vivo* imaging

**Fig. 5:**
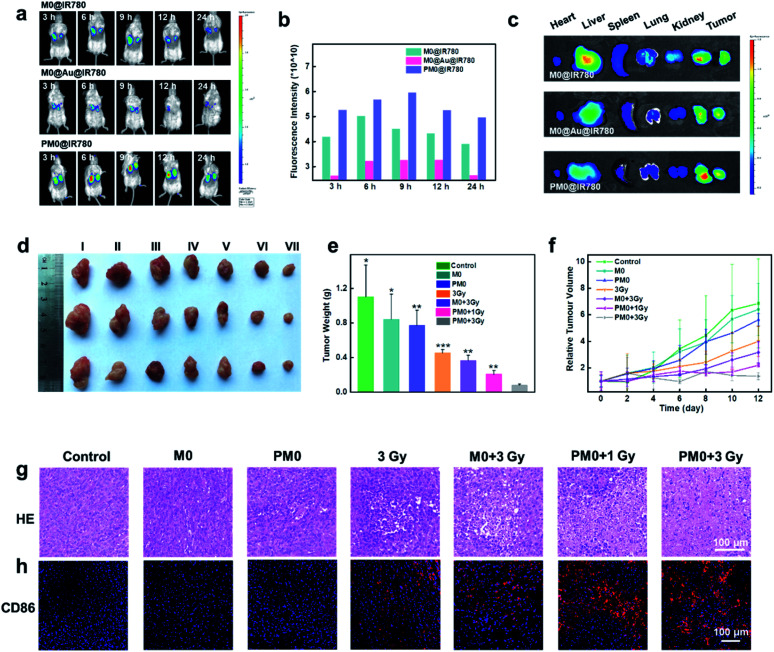
*In vivo* anticancer activity. The *in vivo* fluorescence images of 4T1 tumor-bearing mice at different time points after i.v. injection of M0@IR780, M0@Au@IR780 or PM0@IR780, respectively (a); the corresponding fluorescence intensity of a (b); *ex vivo* fluorescence images of major organs and tumors at 9 h post i.v. injection (c); photographs of representative tumors taken from different groups at 12 days (d); the corresponding tumor weights in different groups (e); tumor growth curves in different treated mice (f); hematoxylin & eosin (H&E)-stained tumor sections in various treated mice (g); immunohistochemical images of CD86+ macrophage in tumor sections of various treated mice (h).

For *in vivo* applications, a bilateral model of 4T1 tumors with different volumes in BALB/c mice was used to investigate the targeting effects of PM0 cells. Macrophages underwent different treatments, were prelabeled with IR780 to obtain the final three cell types (M0@IR780, M0@Au@IR780, PM0@IR780) and then randomly injected (i.v.) into 4T1 tumor-bearing mice (*n* = 3 mice per group). The fluorescence in each group was visualized using an IVIS Imaging System. As shown in Fig. 5a and b, the fluorescence intensity in the two tumor sites was clearly increased at 9 h postinjection with PM0@IR780 and remained strong at 24 h. However, a relatively weak fluorescence signal was detected at 9 h in the other two groups, and the fluorescence intensity decreased quickly. In addition, the imaging system was used to monitor the biodistribution of IR780-labeled macrophages. Major organs and tumors were collected at 9 h, and fluorescence images were captured. As shown in Fig. 5c, mice injected with PM0@IR780 showed stronger IR780 fluorescence intensities in tumors than those in the free macrophage group, indicating much higher macrophage accumulation in tumors after PSA modification. These results demonstrated that the PSA engineering strategy could effectively prolong macrophage circulation in blood and enhance the tumor-targeting of macrophages, and both large- and small-volume tumors could be targeted by PSA-engineered macrophages. The time point of 9 h was chosen for X-ray irradiation administration.

### *In vivo* anticancer activity

*In vivo* therapeutic efficacy was determined using a 4T1 tumor xenograft model. Mice were randomly divided into seven groups and intravenously injected with pretreated macrophages (1 × 10^6^ macrophages per mouse). After 9 h, the mice were given mild X-ray irradiation at a dose of 3 Gy at the tumor site. The tumor volumes and weights of the mice were recorded every 2 days for a total of 12 days. As shown in Fig. 5d–f, mice treated with macrophages or PM0 cells without irradiation displayed significant levels of tumor growth, which was comparable to that in the PBS-treated groups. However, under X-ray irradiation, the amount of tumor regression caused by PBS, macrophages or PM0 cells differed greatly. The PBS group with X-ray irradiation exhibited slight tumor inhibition due to the low radiotherapeutic effects. In contrast, intravenous injection of PM0 cells followed by X-ray irradiation led to effective tumor regression, and the tumor inhibition rate was 67% in response to 1 Gy irradiation and 80% in response to 3 Gy irradiation, while for macrophages exposed to 3 Gy irradiation, the inhibition rate was only 41%. Hematoxylin and eosin (H&E) staining was also performed (Fig. 5g). In the X-ray irradiation groups, the necrotic area in tumor tissues in the PM0+3 Gy group was much larger than that in the other three groups. Furthermore, CD86, a marker of M1 macrophages, was used to evaluate the ratio of M1 macrophages in tumor tissues. As shown in Fig. 5h, the number of CD86-positive cells markedly increased in the PM0+3 Gy group. Additionally, the levels of the proinflammatory cytokines TNF-α and IL-12 were also significantly upregulated in this group (Fig. S8†). Therefore, the enhanced therapeutic efficacy may be attributed to active tumor targeting combined with efficient polarization of macrophages in response to X-ray irradiation. Moreover, there was no obvious damage to normal tissues, as observed by H&E staining (Fig. S9†). The changes in mouse body weight and serum biochemical parameters further confirmed no obvious systemic toxicity in any group (Fig. S10†).

## Conclusions

In summary, we developed a novel X-ray activated phenotype transformation strategy of engineering macrophages for spatiotemporal controlled tumor immunotherapy. M0 macrophages were bio-orthogonally engineered with Au-based PSA. Due to the high density of AS1411 aptamers on PSA, PM0 possess good biocompatibility and prolonged *in vivo* circulation effect. More importantly, the polyvalent binding effect of PSA toward tumor cells endowed PM0 cells with excellent tumor recognition ability. In the *in vivo* antitumor experiments, PM0 was found to target tumors with different tumor volumes more effectively. Under mild X-ray irradiation, ROS generated by Au-based PSA could effectively activate the NF-κB signalling pathway, and PM0 cells could be polarized from the biocompatible M0 phenotype to the antitumoral M1 phenotype *in situ*, thereby showing strong cytotoxicity against tumor cells. As a result, highly efficient tumor accumulation, X-ray-controlled cell phenotype polarization and activatable tumor immunotherapy were realized. PM0 cells significantly inhibited tumor growth even if the irradiation dose was reduced by three-fold compared to the natural macrophage-treated mice plus radiotherapy. All the results confirmed the feasibility of the X-ray-regulated phenotype polarization strategy and proved that X-PT was a promising and safe spatiotemporally manageable approach for clinical tumor immunotherapy.

## Ethical statement

Animal experiments were reviewed and approved by the Ethics Committee of Shandong Normal University, Jinan, P. R. China. All the animal experiments complied with relevant guidelines of the Chinese government and regulations for the care and use of experimental animals.

## Data availability

All experimental supporting data are provided in the ESI.†

## Author contributions

Y. C., P. G., N. L. and B. T. conceived and designed the experiments. Y. C., M. S. and S. L. performed the experiments. Y. C., P. G., W. P., N. L. and B. T. analyzed the data. Y. C., P. G. and W. P. contributed the schematic materials. Y. C., P. G., W. P. N. L. and B. T. co-wrote the paper.

## Conflicts of interest

The authors declare no competing financial interest.

## Supplementary Material

SC-012-D1SC03997K-s001
