# Supplementary material for: Polyvalent spherical aptamer engineered macrophages: X-ray-actuated phenotypic transformation for tumor immunotherapy
Source: Chem Sci. 2021 Sep 22;12(41):13817–24. doi: 10.1039/d1sc03997k (PMC8549783; doi:10.1039/d1sc03997k)
Supplement: SC-012-D1SC03997K-s001 [file SC-012-D1SC03997K-s001.pdf]

Electronic Supplementary Material (ESI) for Chemical Science.
This journal is © The Royal Society of Chemistry 2021

Supplementary Materials for

Polyvalent spherical aptamer engineered macrophages: X-ray-actuated phenotypic transformation for tumor immunotherapy

Yuanyuan Chen, Peng Gao, Wei Pan, Mingwan Shi, Shujie Liu, Na Li,* and Bo Tang*

*Corresponding author. Email: lina@sdnu.edu.cn; tangb@sdnu.edu.cn;

Materials

Tetrachloroaurate acid (III) ($HAuCl_4$•$4H_2O$, 99.99%), sodium dodecylsulfate (SDS), trisodium citrate ($C_6H_5Na_3O_7$•$2H_2O$), Dithiothreitol (DTT) were purchased from China National Pharmaceutical Group Corporation (Shanghai, China). 3-(4,5-dimethyl-thiazol-2-yl)-2,5-diph-enyltetrazolium bromide (MTT) and tris(2-carboxyethyl) phosphine hydrochloride (TCEP) was purchased from Sigma. Glass bottom dishes were purchased from Cellvis (Mountain View, CA). 4T1 cells and 4T1-Luc cells was purchased from Shanghai AOLU Biological Technology Co. Ltd, China. DNA sequences (DNA1 of AS1411 aptamer and DNA2 of SH-T_{60}-DBCO) were synthesized from Sangon Biotechnology Co., Ltd (Shanghai, China) and listed in Table S1. The anti-CD86 antibody, anti-CD80 antibody, the Mouse IL-12 Mini ELISA Kit and Mouse TNF-α ELISA Kit were purchased from Boster Biological Technology Co., Ltd, China. 96-well plates were purchased from Hangzhou Xinyou Biotechnology Co., Ltd, China. Cell membrane tracker (DiO) was purchased from US EVERBRIGHT INC. The chemical reagents were analytical grade and used without any further purification. Sartorius ultrapure water of 18.2 MΩ·cm was used throughout the experiments.

Instruments

Transmission electron microscopy (TEM) was taken on a JEM-100CX II electron microscope. Confocal imaging (CLSM) was acquired with a confocal laser scanning microscopy (Leica TCS SP8, Germany) with an objective lens (×20). Absorption spectra were carried on a TU-1900 UV-Vis spectrometer (Purkinje General, China). Absorbance in the MTT assay was detected using microplate reader (Synergy 2, Biotek, USA). Centrifugation was performed on an Eppendorf 5417R Centrifuge. Fluorescence spectra were recorded with Fluorescence Spectrometer (Edinburgh, FLS-980) with xenon lamp. All pH measurements were measured with a digital pH-3c meter (LeiCi, China).

Synthesis of the AuNPs. The AuNPs with diameter of 13 nm were synthetized according to previous study. $HAuCl_4$ solution (100 mL, 0.01 % w. p.) was heated to 100 °C under vigorous stirring, and then trisodium citrate solution (3.6 mL, 1 % w. p.) was rapidly added into it. When the color changed to burgundy, the colloidal solution was kept boiling for another 20 min and then cooled to room-temperature. Then, the final solution was collected and kept at 4 °C for future use.

Preparation of the polyvalent spherical aptamer (PSA) by freezing method. The solution of DNA1 (2.924 µL, 100 µM) and DNA2 (0.0731 µL, 100 µM) were firstly mixed (DNA1:DNA2 = 40:1). And then, the AuNPs solution (10 nM) added into the mixture. The mixed solution was performed at -20 °C for 60 min and then gradually melted at room temperature. After centrifugation with 12000 rpm for 20 min at 4 °C, the precipitate was obtained and resuspend with deionized water to remove excess DNA. The final PSA were preserved at 4 °C for further use.

Quantitation of each DNA loaded on PSA. The DNA1 and DNA2 loaded on AuNPs were quantitated according to the reported protocol. Firstly, FITC-labeled DNA1 and Cy5-labeled DNA2 were synthetized, and then the dyes-labeled PSA were prepared following the above-mentioned method. Secondly, the dithiothreitol solution (DTT, 20 mM) were mixed with PSA solution (1 nM) and shaken for 24 h. The supernatant solution was collected through centrifugation and the fluorescence of FITC or Cy5 labeled on DNA was measured. The concentrations of DNA1 or DNA2 were calculated based on the standard linear calibration curve. In addition, the number of DNA1 and DNA2 per PSA were also measured via nanodrop.

Cell culture. The 4T1 cells used in the experiments were treated with 1640 medium containing 10% fetal bovine serum and 1% 100 U/mL penicillin/streptomycin. The macrophages (Raw264.7 cells) used in the experiments were treated with DMEM medium containing 10% fetal bovine serum and 1% 100 U/mL penicillin/streptomycin. Cells were incubated in a humidified atmosphere with 5% CO_2, 95% air at 37 °C.

Cytotoxicity of Ac_4ManNAz or PSA. The cytotoxicity of Ac_4ManNAz or PSA was performed *via* the MTT assay to macrophage. Macrophage were dispersed within replicate 96-well microtiter plates and were maintained at 37 °C in a 5% CO_2 / 95% air incubator for 24 h. 1) Different concentration of Ac_4ManNAz (0, 25, 50, 80, 100 µM) were added into cells, respectively. 2) Different concentration of PSA (0, 0.25, 0.50, 1.00, 1.50 nM) were added into cells, respectively. After incubation for another 24 h, the cell medium removed and MTT solutions (0.5 mg/mL, 150 µL) were added to each well. Later, DMSO (150 µL) was added to dissolve the formazan crystals. The absorbance at 490 nm was measured with a synergy 2 microplate reader.

Influence of macrophages with different treatment. 1) Macrophage treated with Ac_4ManNAz: Macrophage were divided into three group and suffered with different treatment, respectively (PBS, Ac_4ManNAz (50 µM) for 24 h, Ac_4ManNAz (50 µM) for 24 h and then $MnCl_2$ for another

24 h. 2) Macrophages treated with PSA: Macrophages were divided into three group and suffered with different treatment, respectively (PBS, PSA (1.0 nM) for 2 h, PSA (1.0 nM) for 2 h and then $MnCl_2$ for another 24 h). After that, cells were collected and stained with FITC anti-mouse CD80. Finally, an imaging flow cytometer (Amnis Corporation) was applied to record the cell images with an excitation of 488 nm for FITC. IDEAS® image analysis software (Amnis) was used to analyze the images.

Generation of PSA-equipped M0 macrophage (PM0) *via* a straightforward chemical strategy. To obtain PM0, macrophages were supplied with Ac_4ManNAz (50 μM) for 24 h and washed with PBS for 3 times. After that, the cells were further incubated with DBCO-decorated PSA (1.0 nM) in DMEM supplemented with 1% FBS for 2 h. And then, the cells were washed with PBS buffer for 3 times and imaged by dark-field microscopy (DFM) and confocal laser scanning microscopy (CLSM).

The tumor cells-targeting of PM0. 1) The targeting of PSA: cy5-labled PSA (1 nM) was added into the 4T1 cells or TC-1 cells, respectively. After incubation for 1 h, cells were washed with PBS for three times and stained with Hoechst for 15 min. And then, cells were imaged by CLSM. 2) The targeting of PM0： Firstly, PM0 were labeled with DiO for 10 min and 4T1 cells were labeled with Hoechst for 15 min, respectively. After washed with PBS for three times, cell suspension of PM0 and 4T1 cells were mixed with the radio of 2:1 and then shaken for 1 h at 37 °C. Cells were put in in confocal dishes and then imaged by CLSM. Macrophages (green) and 4T1 cells (blue) were counted respectively. The percentage of captured 4T1 cells was reported as the ratio of adherent blue cells to total blue cells. 3) 4T1 cells were incubated in confocal dishes for 24 h and then labeled with Hoechst for 15 min. The suspension of PM0 prelabeled with DiO for 10 min were added into the confocal dishes. After coincubation for 1 h, the unbound PM0 subsequently removed and cells were imaged by CLSM.

Stimulated differentiation of PM0. To observe the phenotypic transformation of macrophages, the macrophages treated with different conditions was investigated (Control, 1 Gy, 3 Gy, PSA+1 Gy, PSA+3 Gy). The macrophages were stained with FITC-labeled anti-CD80 antibody or PE-labeled anti-CD86 antibody and then visualized under CLSM or flow cytometry analysis.

The mechanism of M0-to-M1 repolarization. 1) ROS detection: macrophages were divided into five groups and treated with different conditions (Control, 1 Gy, 3 Gy, PSA+1 Gy, PSA+3 Gy).

The cells were stained with DCFH-DA and further visualized by flow cytometry analysis. 2) Intracellular NF-κB measurement: The cells were given different treatment. After further incubation for 12 h, the cells were fixed with paraformaldehyde (4%) for 15 min, and then incubated with primary antibody of NF-κB p65 rabbit mAb for 1 h and FITC-labeled secondary antibody for another 1 h at room temperature, respectively. The cells were further labeled with Hoechst for 15 min. Finally, the cells were washed with PBS for CLSM analysis. In addition, the expression of NF-κB and p-MAPK in cells of each group were also performed by western blotting analysis.

Quantification of cytokine secretion. To assess the changes of cytokines secreted by PM0, cells were seeded into a 6-well plate for 24 h and then given different treatment (Control, 1 Gy, 3 Gy, PSA+1 Gy, PSA+3 Gy). After the cells further incubated for 24 h, the supernatant was collected by centrifugation and further evaluated by the ELISA Kit (R&D) according to the manufacturer's protocols.

The enhanced cytotoxicity of PM0. 1) Nitric oxide (NO) detection: Firstly, a fluorogenic probe for NO detection was prepared according to previous study. Secondly, macrophages were divided into five groups and treated with different conditions (Control, 1 Gy, 3 Gy, PSA+1 Gy, PSA+3 Gy). After incubation for another 12 h, the culture medium was collected with further centrifugation. Finally, the NO concentration in each group was detected. 2) The chemotaxis of macrophage was investigated using a Transwell migration assay. The lower compartment was previously incubated with 4T1 cells for 24 h. Macrophages were divided into five groups and treated with different conditions. After incubation for 12 h, cells were suspended in DMEM medium and plated in the upper chamber of the Transwell. Followed co-incubating for 6 h at 37 °C, cells migrating across the Transwell in the lower chamber were stained with crystal violet and detected under microscope. 3) Measurement of the efficiency of killing 4T1 cells with culture medium of macrophages. 4T1 cells were firstly transferred into a 24-well plate for incubation 24 h. Macrophages were treated differently according to the mentioned above and then seeded in the upper Transwell chamber (0.4 μm) in 24-well plate, respectively. After coincubation for 6 h, MTT assay was carried out to detect cell viability of 4T1. 4) Measurement of the efficiency of killing 4T1 cells of macrophages. 4T1 cells were firstly transferred into a 96-well plate for incubation 24 h. Macrophages were treated differently according to the mentioned above and then seeded in the

96-well plate, respectively. Meanwhile, equivalent macrophages were also seeded in the 96-well plate, respectively, where no 4T1 cells was supplied in the chamber. After coincubation for 6 h, MTT assay was carried out to detect cell viability of 4T1 cells.

Mice culture. Animal experiments were reviewed and approved by the Ethics Committee of Shandong Normal University, Jinan, P. R. China. All the animal experiments complied with relevant guidelines of the Chinese government and regulations for the care and use of experimental animals.

***In vivo* Imaging.** Firstly, a bilateral model of 4T1 tumors on BALB/c mice were employed to investigate the targeting effect of the cells. BALB/c mice were subcutaneously (s.c.) injected with 1×10^6 4T1 cells into the right flank and 1×10^4 4T1 cells injected into the left flank, respectively. Secondly, macrophages with different treatment were prelabeled with IR780 to obtain the final three cells (M0@IR780, M0@Au@IR780, PM0@IR780). Finally, the labeled cells were intravenously injected (i.v.) into 4T1 tumor-bearing mice. The fluorescence in each group was visualized using an IVIS Imaging System (PerkinElmer). At 9 h after injection, the mice were sacrificed, and major organs (heart, lung, liver, kidney, and spleen) and tumors were dissected from the mice to further investigate the biodistribution.

***In vivo* tumor therapy.** BALB/c mice were subcutaneously (s.c.) injected with 1×10^6 4T1 cells into the right flank. When the tumor volume reached about 50 mm^3, the mice were randomly divided into 7 groups. The mice were intravenously injected with (1) PBS, (2) M0, (3) PM0, (4) PBS, (5) M0, (6) PM0, (7) PM0, respectively. After 9 h injection, the mice in (4), (5) and (7) were irradiated with the dose of 3 Gy and the mice in (6) were irradiated with the dose of 1 Gy. The tumor volume was monitored for 12 days and calculated by formula: $V=L\times W\times W/2$ (L, the longest dimension; W, the shortest dimension). The body weight of each mouse was also monitored.

Potential toxicity assessment. At 24 h after treatment, major organs and tumors were collected for hematoxylin and eosin (H&E). At three days after treatment, the tumors slices were collected and the immunofluorescence assays for M1 macrophages was performed by staining with CD86. Meanwhile, the serums were collected by centrifugation and the changes of cytokines secreted by mice were further evaluated by the ELISA Kit according to the manufacturer's protocols. For safety evaluation, the serum levels of alkaline phosphatase (ALP), alanine aminotransferase (ALT), aspartate transaminase (AST), blood urea nitrogen (BUN), creatinine (Cr) were analyzed.

At 7 days after treatment, major organs and tumors were collected for H&E staining to further examine histological changes and assess the long-term systemic toxicities.

Supplementary Figures

Name	sequences
DAN1	SH-C6-TTTTTTTTTGGTGGTGGTGGTTGTGGTGGTGGTGGT
DNA1-FAM	SH-C6-TTTTTTTTTGGTGGTGGTGGTTGTGGTGGTGGTGGT-6-FAM
DNA2	HS-SH-C6 TTTTTTTTTTTTTTTTTTTTTTTTTTTTTTTTTTTTTTTTTTTTTTTTTTTT TTTTTTT-DBCO
DNA2-Cy5	HS-SH-C6 TTTTTTTTTTTTTTTTTTTTTTTTTTTTTTTTTTTTTTTTTTTTTTTTTTTT TTTTTTT-Cy5

TableS1 DNA sequences

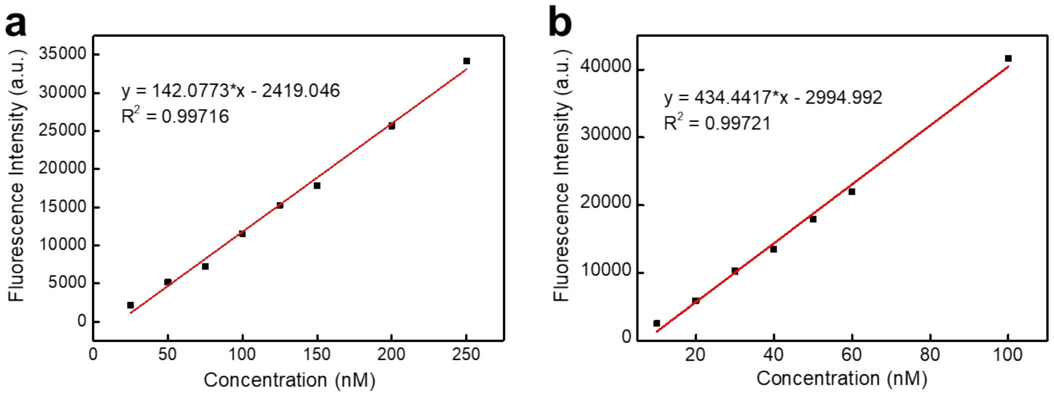

Fig. S1. Standard linear calibration curve of DNA1-FAM (a) and DNA2-Cy5 (b).

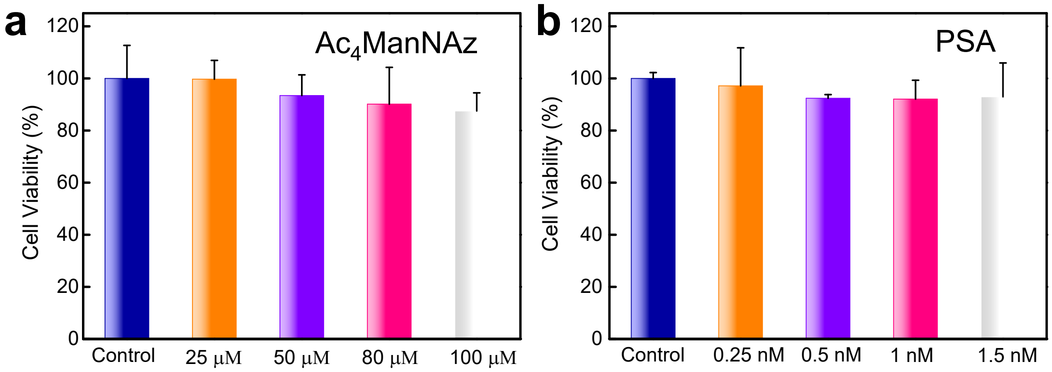

Fig. S2. The cytotoxicity assays of Ac$_4$ManNAz (a) or PSA (b) on M0 cells after 24 h incubation.

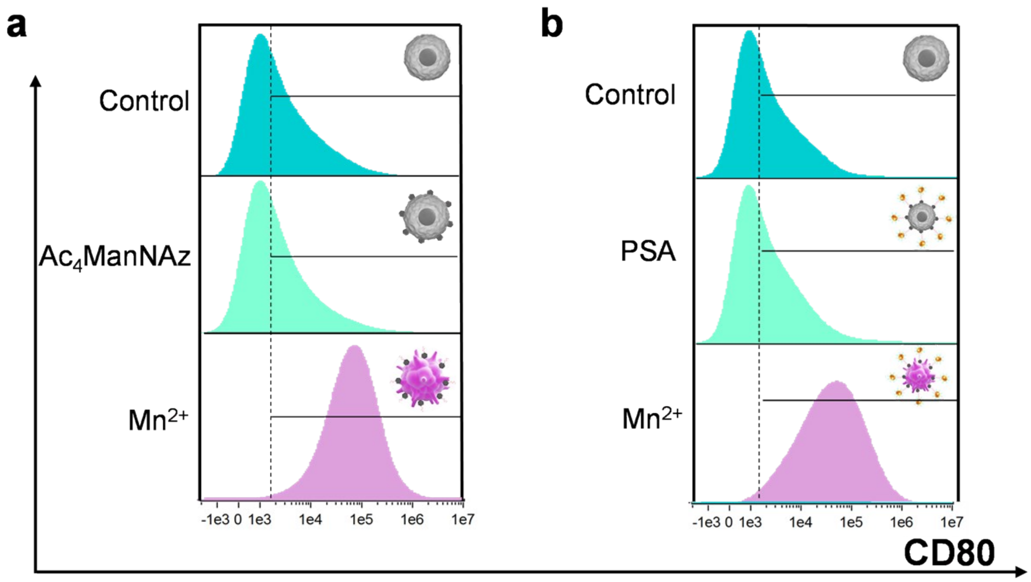

Fig. S3. Flow cytometry analysis of CD80 expression (M1 marker) on macrophage after treatment with different condition. Macrophage were co-incubated with Ac$_4$ManNAz (50 mM) for 24 h and further treated with Mn^{2+} (0.2 mM) for another 24 h (a); Macrophage were co-incubated with PSA (1 nM) for 2 h and further treated with Mn^{2+} (0.2 mM) for another 24 h (b);

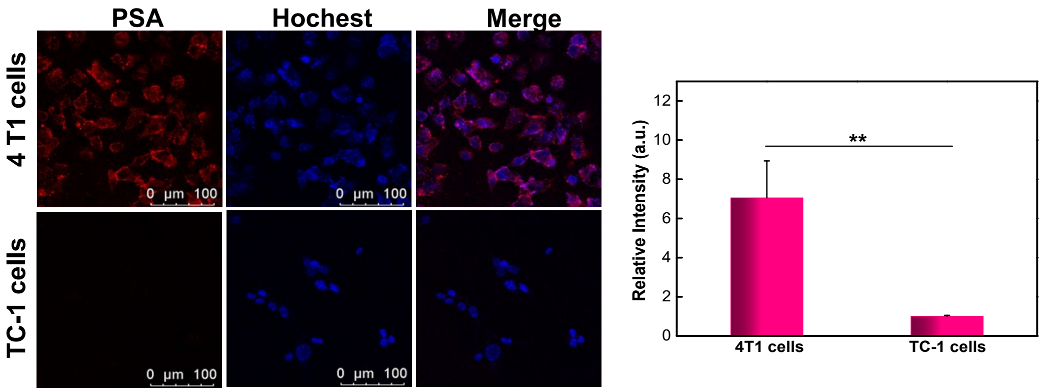

Fig. S4. Effect of AS1411 aptamer on the cancerous (4T1 cells) and the normal cells (TC-1 cells) (a); The corresponding relative intensity of a (b);

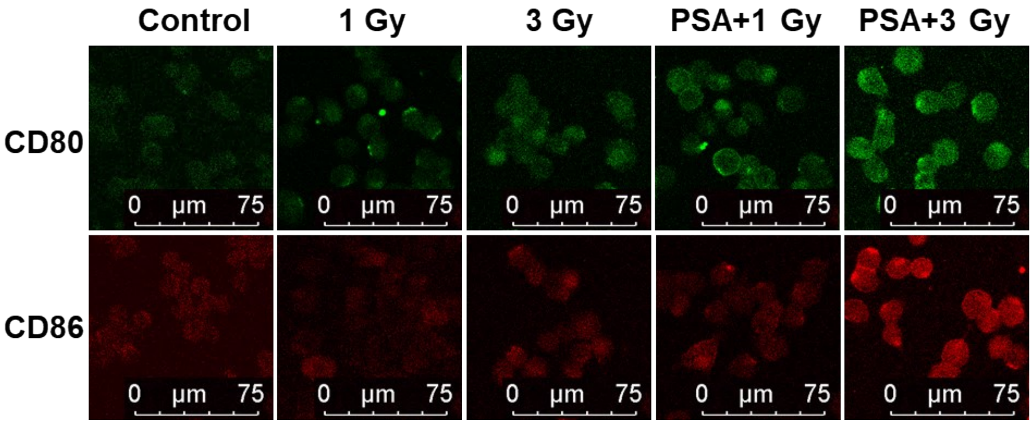

Fig. S5. Immunofluorescence imaging of the CD80 and CD86 expression in macrophages after treatment with different conditions.

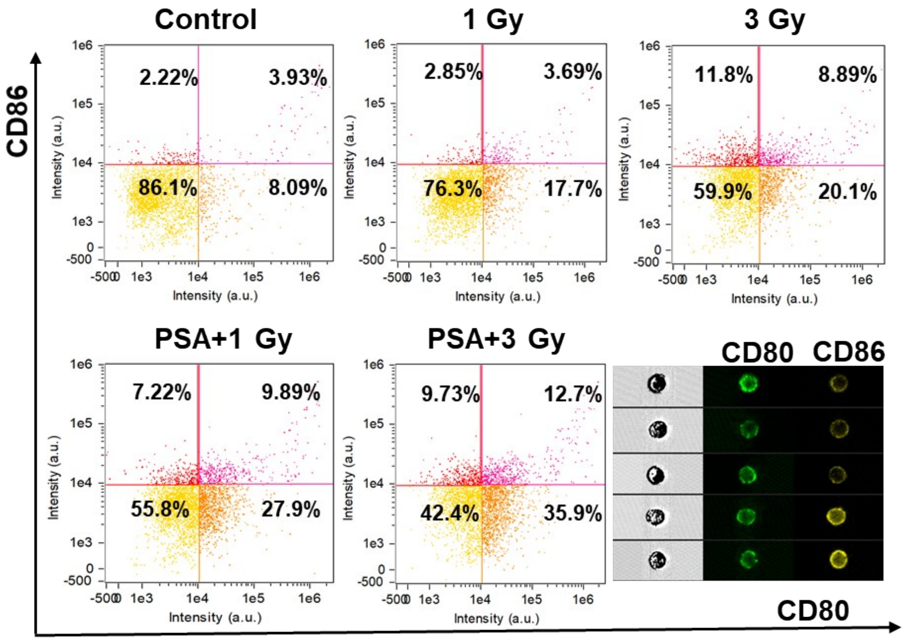

Fig. S6. Flow cytometry analysis of the CD80 and CD86 expression in macrophages after treatment with different conditions.

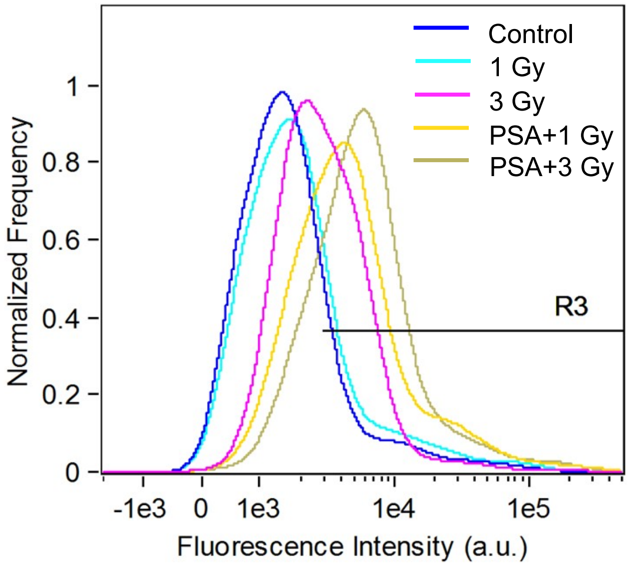

Fig. S7. Flow cytometry analysis of intracellular ROS generation in different treated macrophages.

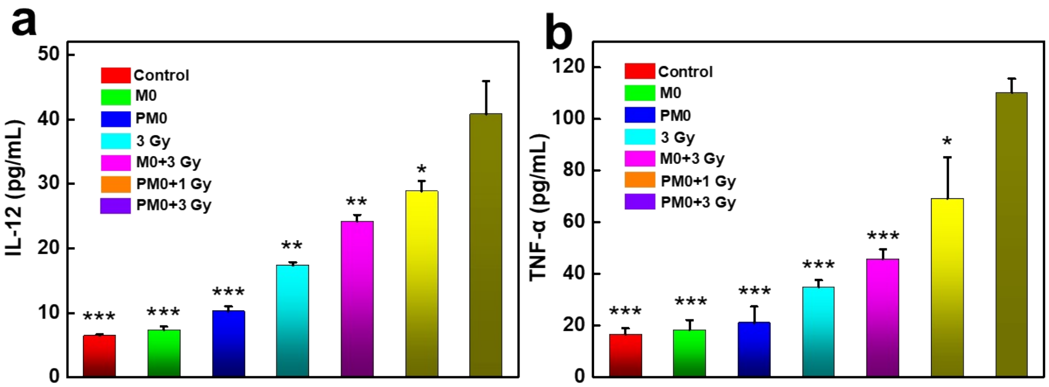

Fig. S8. Serum immune cytokines concentrations of IL-12 (a) and TNF-α (b) detected by ELISA kit from the mice before sacrifice.

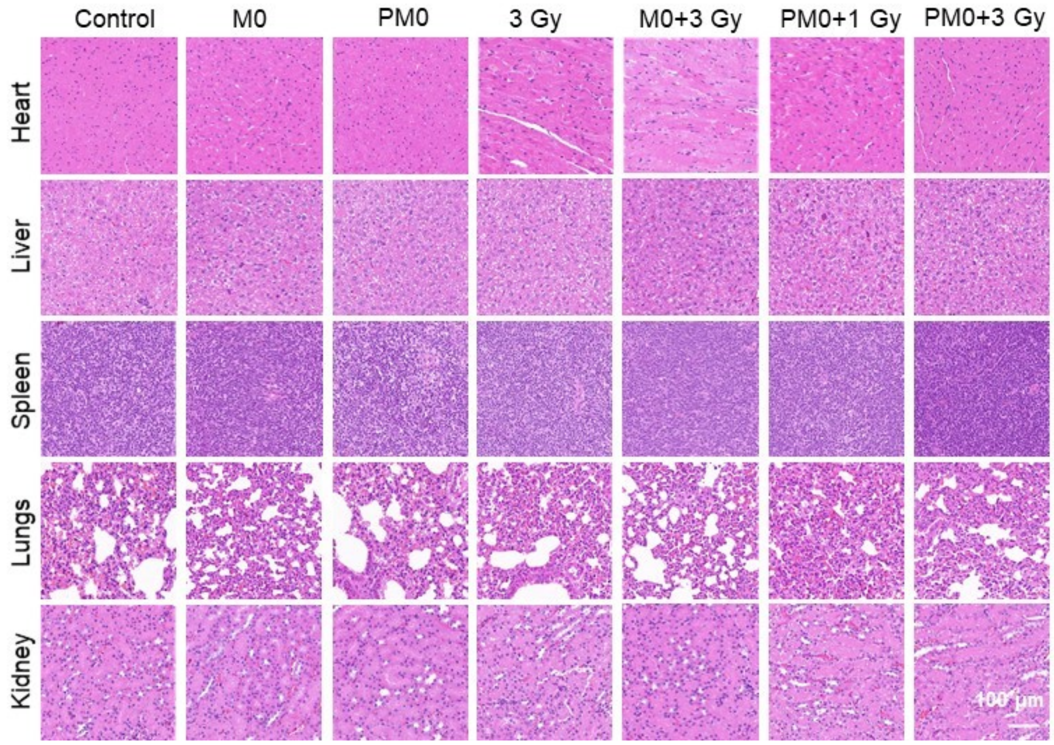

Fig. S9. H&E staining of the lung, liver, spleen, heart and kidney after different treatment.

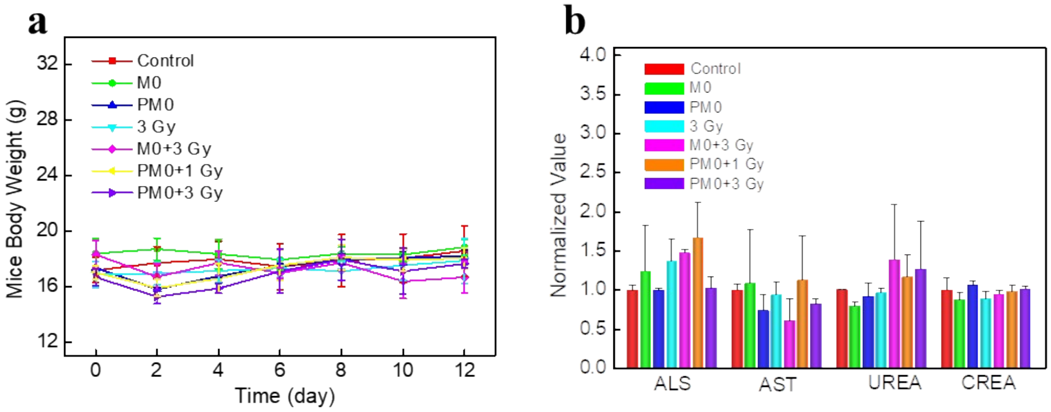

Fig. S10. The mice bodyweight curves in different treated groups of tumor-bearing mice (a); Histograms of the hematological analysis of ALT, AST, UREA, CREA in mice after different treatment (b).
